# Co-Encapsulation of Fisetin and Cisplatin into Liposomes for Glioma Therapy: From Formulation to Cell Evaluation

**DOI:** 10.3390/pharmaceutics13070970

**Published:** 2021-06-26

**Authors:** Morgane Renault-Mahieux, Victoire Vieillard, Johanne Seguin, Philippe Espeau, Dang Tri Le, René Lai-Kuen, Nathalie Mignet, Muriel Paul, Karine Andrieux

**Affiliations:** 1 Unité de Technologies Chimiques et Biologiques pour la Santé (UTCBS), Centre National de la Recherche Scientifique (CNRS), Institut National de la Santé et de la Recherche Médicale (INSERM), Université de Paris, 4 Avenue de l’Observatoire, 75006 Paris, France; morgane.renault@aphp.fr (M.R.-M.); johanne.seguin@u-paris.fr (J.S.); philippe.espeau@u-paris.fr (P.E.); ledangtri@hotmail.com (D.T.L.); nathalie.mignet@u-paris.fr (N.M.); 2 Henri Mondor Hospital Group, Pharmacy Department, Assistance Publique—Hôpitaux de Paris (AP-HP), 51 Avenue du Maréchal de Lattre de Tassigny, 94010 Créteil, France; victoire.vieillard@aphp.fr (V.V.); muriel.paul@aphp.fr (M.P.); 3 UMS3612 Centre National de la Recherche Scientifique (CNRS), US25 Institut NATIONAL de la Santé et de la Recherche Médicale (INSERM), Plateforme Mutualisée de l’Institut du Médicament (P-MIM), Plateau Technique Imagerie Cellulaire et Moléculaire, Université de Paris, 75006 Paris, France; rene.lai-kuen@u-paris.fr

**Keywords:** liposomes, fisetin, cisplatin, co-encapsulation, glioblastoma

## Abstract

(1) Background: Glioblastoma (GBM) is the most frequent cerebral tumor. It almost always relapses and there is no validated treatment for second-line GBM. We proposed the coencapsulation of fisetin and cisplatin into liposomes, aiming to (i) obtain a synergistic effect by combining the anti-angiogenic effect of fisetin with the cytotoxic effect of cisplatin, and (ii) administrate fisetin, highly insoluble in water. The design of a liposomal formulation able to encapsulate, retain and deliver both drugs appeared a challenge. (2) Methods: Liposomes with increasing ratios of cholesterol/DOPC were prepared and characterized in term of size, PDI and stability. The incorporation of fisetin was explored using DSC. The antiangiogneic and cytotoxic activities of the selected formulation were assayed in vitro. (3) Results: We successfully developed an optimized liposomal formulation incorporating both drugs, composed by DOPC/cholesterol/DODA-GLY-PEG2000 at a molar ratio of 75.3/20.8/3.9, with a diameter of 173 ± 8 nm (PDI = 0.12 ± 0.01) and a fisetin and cisplatin drug loading of 1.7 ± 0.3% and 0.8 ± 0.1%, respectively, with a relative stability over time. The maximum incorporation of fisetin into the bilayer was determined at 3.2% *w*/*w*. Then, the antiangiogenic activity of fisetin was maintained after encapsulation. The formulation showed an additive effect of cisplatin and fisetin on GBM cells; (4) Conclusions: The developed co-loaded formulation was able to retain the activity of fisetin, was effective against GBM cells and is promising for further in vivo experimentations.

## 1. Introduction

Glioblastoma (GBM) is the most frequent primary brain tumor. It is an aggressive astrocytic tumor (grade IV in the WHO designation) associated with a median overall survival of 12 months after standard therapy and a relative survival of 8.8% at three years and only 5.1% at five years (6.3% in Europe) [1,2]. The standard treatment of GBM consists of surgery associated with radiotherapy and chemotherapy with temozolomide, a prodrug of a cytotoxic agent able to alkylate DNA [3]. However, there are no guidelines for the treatment of relapses. Amongst several options [4], anti-angiogenic therapy is an emerging and promising strategy because of the high vascularization in GBM. The angiogenesis mechanisms of these tumors are related to multiple possible genetic alterations of the angiogenesis pathway and to the hypoxic environment of the tumor leading to a microvascular proliferation [5]. However, anti-angiogenic therapy alone does not seem to be sufficient and there is a need for combinational therapy with chemotherapy [6]. In fact, bevacizumab, an anti-VEGF antibody, is already approved by the FDA for the treatment of recurrent GBM in combination with irinotecan, a topoisomerase 1 inhibitor [7,8].

Fisetin (3,3′,4′,7-tetrahydroxyflavone) is a natural flavonoid present in several fruits and vegetables. This hydrophobic molecule possesses several biological effects such as antioxidant, anti-inflammatory, and antiangiogenic [9,10]. Its capacity to stabilize endothelial cell microtubules [11,12] and to improve antiangiogenic effect and anticancer activity in mice bearing Lewis lung carcinoma in association with cyclophosphamide has been demonstrated [13,14]. Moreover it could inhibit the human glioma cancer cell invasion in vitro [15]. However, due to its physicochemical properties, fisetin cannot be administered alone; it should either be solubilized into organic solvent, which would not be appropriate, or encapsulated in a drug delivery system. Delivery of polyphenols is particularly challenging [16]; however, interesting results were previously obtained upon fisetin encapsulation into liposomes or nanoemulsions [17,18]. Therefore, encapsulation of fisetin into a nanocarrier could overcome this limitation.

Cisplatin is a relatively water-soluble alkylating agent approved by the FDA and the EMA in several cancer treatments. It has been proven that cisplatin was efficient in vitro on GBM cell lines [19,20,21] or in vivo administered locally [22]. However, systemic administration of cisplatin led to disappointing results with an increased toxicity, underlining the difficulty of cisplatin to attain the GBM site [23,24,25]. The side effects of cisplatin are due to its ability to form covalent adducts with DNA. Cisplatin is well known for its nephrotoxicity, neurotoxicity, and its capacity to induce nausea and vomiting, but also ototoxicity and cardiotoxicity [26]. Encapsulating cisplatin into a nanocarrier could improve the benefit/risk ratio by increased accumulation of the drug into the tumor using the EPR (enhanced permeability and retention) effect and decreased distribution into other tissues [27,28].

Combinational therapy using nano-structures delivery has been extensively investigated for cancer therapy [29,30]. A liposomal formulation encapsulating both anticancer drugs daunorubicin and cytarabin (CPX-351, Vyxeos^®^, Jazz Pharmaceuticals, Dublin, Ireland) for the treatment of acute myeloid leukemia was approved by the FDA in 2017 and EMA in 2018 [31]. Moreover, several drugs loaded in liposomal formulations and administered intravenously or with an intracerebral route have been used in several clinical trials for the treatment of central nervous system diseases and have been able to reach their target, showing increased efficacy and reduced toxicity [32].

In this work, a novel combinatory approach against GBM is envisioned with the aim to obtain a synergistic effect of the antiangiogenic fisetin and the anticarcinogenic cisplatin. To enhance fisetin solubility and combine both activities, an encapsulation of the two drugs seemed compulsory. Liposomes are vesicles composed of an aqueous core encountered by a lipidic bilayer. Liposomes due to their specific core/shell structure are promising candidates to co-encapsulate a hydrophilic drug such as cisplatin in its aqueous core and fisetin, a hydrophobic molecule, into the lipid bilayer. In a previous work, our group designed a liposomal formulation of fisetin with a low content of cholesterol to provide high fisetin content [17]. Other authors have described cisplatin-loaded liposomes with a high content of cholesterol in order to limit its rapid release [33,34,35,36]. The challenge here was to design a liposomal formulation able to encapsulate and retain both drugs.

## 2. Materials and Methods

### 2.1. Reagents

Fisetin (98% purity) was purchased from Shanghai FWD Chemicals Limited (Shanghai, China), cisplatin, cholesterol (Chol), acetonitrile (ACN), methanol, HEPES, PBS (phosphate buffer saline), triton X-100 solution from Sigma-Aldrich (Saint-Louis, MO, USA) and dioleyl-phosphatidylcholine (DOPC) and 1,2-distearoyl-*sn*-glycero-3-phosphocholine (DSPC) from Polar Avanti Lipids, Inc. (Coger, Paris, France). Chloroform, absolute ethanol and sodium hydroxide 1N were purchased from Carlo Erba Reagents (Val de Reuil, France), acetic acid (98–100%) and nitric acid 65% from Merck Millipore (Darmstadt, Germany), hydrochloric acid 37% from VWR Chemicals (Fontenay-sous-Bois, France) and sodium chloride from Cooper (Melun, France). Dubelcco’s modified Eagle’s medium (DMEM), Eagle’s minimal essential medium (EMEM), fœtal bovine serum (FBS), streptomycin and penicillin were purchased from Thermo Fisher Scientific (Waltham, MA, USA). Deionized water was obtained using a Direct-Q3 device from Millipore. (2-dioctadecylcarbamoyl-methoxyacetylamino) acetic acid-(ω-methoxy)-polyethylene glycol 2000 ester (DODA-GLY-PEG2000) has been synthesized as described before [17].

### 2.2. Preparation of Liposomes

The process, except the extrusion, was carried out in a heating bath at 40 °C. All liposomes were prepared using the film-hydration method [37]. Briefly, lipids with or without fisetin were dissolved in a chloroform–ethanol mixture and introduced in a clean dry round-bottom flask and evaporated by a Buchi evaporator R-100 equipped with a V-100 pump and a F-105 recirculating cooler. The mixture was exposed to a starting pressure at 250 mbar and then 100 mbar to form a regular film on the bottom. The pressure was then decreased to reach 10 mbar, which was maintained for three hours. HEPES buffer (20 mM, pH = 7.4) with or without cisplatin dissolved in it was added to the flask and the rehydration process was performed overnight, providing an aqueous suspension of liposomes.

The size calibration was performed using extrusion of the liposomes through 0.4 and 0.2 µm polycarbonate membranes (Whatman) three times each using a Mini-extruder (Avanti Polar Lipids, Inc., Alabaster, AL, USA) at room temperature for the DOPC liposomes and 65 °C for the DSPC liposomes.

### 2.3. Purification of Liposomes

In order to remove non-encapsulated fisetin or cisplatin, a purification step is necessary. Fisetin-loaded liposomes were either purified using filtration under vacuum through a 0.45 µm filter (Sartorius, Göttingen, Allemagne) or using size-exclusion chromatography on Sephadex G-25 columns (GE Healthcare, Chicago, IL, USA). Cisplatin-loaded liposomes and co-loaded liposomes purification were carried out using size-exclusion chromatography on Sephadex G-25 columns (GE Healthcare). For DSC experiments, there was no purification step.

### 2.4. Formulation Tested

A formulation of fisetin-loaded liposomes composed of DOPC/cholesterol/DODA-GLY-PEG2000 (87.0/8.9/4.1 molar ratio) was described in our previous work [17] (F1, Table 1). Several formulations of liposomes with an increasing cholesterol/DOPC ratio were prepared as described above. All their lipid compositions are reported in Table 1. As the formulation names refer to the lipid composition and the study aims to study the influence of lipid composition on the encapsulation of both drugs, the authors have chosen to use the same name with mention made to the drug encapsulated or co-encapsulated.

### 2.5. Characterization of Liposomes

#### 2.5.1. Determination of Size and Morphology

Dynamic light scattering (DLS): the liposome’s diameter and the polydispersity index (PDI) were determined after dilution in HEPES buffer at a lipid concentration of approximatively 0.5 mg/mL by DLS using a Zeta Sizer NanoSeries Malvern (Malvern Instrument, Venissieux, France) at 25 °C with a measurement angle of 173°. The position was fixed at 4.65 cm. The results presented in this article correspond to the diameter calculated by using the intensity of the signal.

Transmission electronic microscopy (TEM): a liposomal suspension was deposed on a Formvar/carbon copper grid 200 mesh from Agar Scientific and uranyl acetate as a contrasting agent. TEM observations were performed on a microscope JEOL, JEM 100S with an accelerating voltage of 80 kV.

#### 2.5.2. Fisetin Quantification

Fisetin was assayed by high-performance liquid chromatography after purification using a reverse phase HPLC system (Dionex U3000, Thermo Fisher Scientific, Waltham, MA, USA) equipped with a polymeric PRP-1 250 × 4.6 mm, 5 µm (Hamilton Company, Reno, NV, USA). The injected volume was 10 µL. Eluent A consisted of 2% acetic acid in deionized water and eluent B was ACN. The flow rate was 1 mL/min. The elution gradient started with 15% B for 3 min. From 3 to 6 min, the percentage of eluent B was increased to achieve 50%, which was maintained for 6 min, to turn back to 15% in 2 min, followed by an equilibrium phase of 2 min. The UV detection of the drugs was carried out at 360 nm as it is the maximum absorbance of fisetin determined by spectrophotometry. Data were processed using the Chromeleon^®^ (v6.8) software (Thermo Fisher Scientific, Waltham, MA, USA).

The calibration curve was prepared by dissolving fisetin in methanol. This method was validated according to ICH Q2 (R1) (Peak area = 0.177 × Concentration + 0.851, R² = 0.999 ± 0.001). The purified fisetin-loaded or liposomes co-encapsulating fisetin and cisplatin were diluted in methanol prior to the injection to range in the calibration curve (5–100 µg/mL).

#### 2.5.3. Cisplatin Quantification

Cisplatin was assayed after purification against a standard curve (300–2500 ng/mL) using a Zeeman Atomic Absorption Spectrometer AA240Z (Agilent Technologies, Santa Clara, CA, USA) with a graphite tube atomizer (GTA120) connected to a programmable sample dispenser (PSD120) as previously described [38]. The detection was monitored at 265.9 nm with a slit band width of 0.2 nm (lamp current fixed at 10 mA). The program consisted of a drying stage from 50 to 250 °C, an ashing stage at 1400 °C, an atomization phase at 2700 °C and a burning-clean stage at 2700 °C with cooling down at 50 °C. The standard solutions were prepared using a solution of cisplatin 3.07 µg/mL in HEPES buffer 20 mM pH = 7.4 mixed with a suspension of blank liposomes; this stock solution was directly diluted by the autosampler with an acidic buffer (hydrochloric acid 0.6% in physiological serum). A matrix modifying solution of Triton X-100 0.1% + nitric acid 0.2% was applied to each standard solution and sample. Samples were diluted with the acidic buffer to fit in the range of the calibration range. This method was validated according to ICH Q2 (R1) (quadratic regression, R^2^ = 0.9999).

#### 2.5.4. Determination of Encapsulation Efficiency and Drug Loading

The encapsulation efficiency (EE) was determined as:

EE (%, *w*/*w*) = (drug concentration after purification/total amount of drug in the formulation) × 100.

Drug loading (DL) was determined as:

DL (%, *w*/*w*) = (drug concentration after purification/(concentration of lipids + drug concentration after purification)) × 100.

The drug-to-lipids ratio, corresponding to the amount of drug (mg) per total lipid amount (g), was also calculated for fisetin.

#### 2.5.5. Differential Scanning Calorimetry

Thermal analyses were conducted by differential scanning calorimetry (DSC), using a Mettler Toledo 822 DSC (Greifensee, Switzerland) calibrated beforehand using high-purity indium (99.99%, provided by Mettler-Toledo; temperature and enthalpy of fusion: Tfus = 156.6 °C and Δ_fus_H = 28.45 J g^−1^, respectively). Measures of 10 µL of samples were injected in aluminum standard pan of 40 µL and hermetically sealed. An empty hermetically sealed aluminum pan was used as reference. Since DOPC constituted the major component of the liposomal bilayer; we investigated the interaction of DOPC with fisetin and cholesterol inserted into the bilayer based on the thermal events of DOPC during the melting process (Tm ≈ −18 °C). The experiments were carried out by a decrease in temperature from 25 to −80 °C and an increase from −80 to 140 °C with a heating rate of 10° K/min. Data analyses were performed with the programs provided by the constructor.

### 2.6. Stability Studies

Drug encapsulation into liposomes was followed as a function of time. Liposomal suspensions were stored at 5 ± 3 °C. Samples were withdrawn, purified again and characterized at each sampling time. The percentage of each drug leakage during storage was calculated as:

100 − (concentration of drug still encapsulated at day of sampling/EE at day 0) × 100.

### 2.7. Formulation Optimisation

#### 2.7.1. Impact of the Cholesterol Ratio

Formulations 3, 4 and 6 (Table 1) were chosen to incorporate either fisetin, cisplatin or both drugs. Formulations 3, 4 and 6 were prepared and characterized as described in Section 2.2 and Section 2.5 with the addition of fisetin in the lipid phase in order to obtain a theoretical concentration of 1.2 mg/mL in the final suspensions for fisetin-loaded liposomes, and with the addition of cisplatin at a concentration of 2 mg/mL in HEPES buffer for cisplatin-loaded liposomes. The liposomal suspensions were compared in term of size, PDI, DL and stability over 10 days.

#### 2.7.2. Impact of the Saturation of the Phospholipids

Fisetin-loaded liposomes using DSPC instead of DOPC (F2 and F5, Table 1) were prepared and extruded at 65 °C. The liposomal suspensions were compared in term of size, PDI, DL and stability over 10 days.

#### 2.7.3. Impact of the Co-Encapsulation

Formulations 3 and 4 (Table 1) were prepared as described above, with the addition of fisetin in the lipid phase in order to obtain a theoretical concentration of 1.2 mg/mL in the final suspensions and with the addition of cisplatin at a concentration of 2 mg/mL in HEPES buffer. The liposomal suspensions were compared in term of size, PDI, DL and stability over 10 days.

#### 2.7.4. Impact of the Initial Fisetin Amount

First, formulations with an increasing ratio of cholesterol/DOPC and a fixed amount of fisetin (3.2% *w*/*w*) were prepared and analyzed using DSC, as described in Section 2.5.5. The enthalpies and onset temperatures of the transition peaks were compared. Then Formulation F4 was chosen to investigate the incorporation of fisetin inside the lipid bilayer. Liposomes were prepared with different amounts of fisetin ranging from 0 to 26.5 mol% (0 to 11.8% *w*/*w*) in deionized water and analyzed using DSC, as described in Section 2.5.5.

F4 liposomes with fisetin ranging from 1 to 4.5% *w*/*w* of the liposomal bilayer were also prepared in HEPES buffer and purified using filtration under vacuum through a 0.45 µm filter (Sartorius). Their EE, DL and stability over 30 days were investigated.

### 2.8. In Vitro Release

The release of fisetin and cisplatin from the co-loaded liposomes in phosphate buffer saline (PBS) and in DMEM containing 2 mg/mL glutamine, 10% FBS, 100 µg/mL streptomycin and 100 UI/mL penicillin (DMEM-C) (viscosity 0.94 cP) was investigated over 48 h. The samples were put in a dialysis bag CelluSep H1 with a cut-off of 25,000 Da (Dutscher, France) with a volume of sample to volume of receiving medium ratio fixed at 1:100. The dialysis bag kept floating in receiving medium at 37 °C with an agitation rate of 90 rpm. At each sampling time, the liposomal suspension was removed and fisetin and cisplatin were extracted and assayed as described in Section 2.5.2 and Section 2.5.3.

### 2.9. Colloidal Stability in Culture Medium

F4 co-loaded liposomes were prepared and diluted 40-fold in DMEM containing 2 mg/mL glutamine, 10% FBS, 100 µg/mL streptomycin and 100 UI/mL penicillin (DMEM-C) (viscosity 0.94 cP) and kept at 37 °C for 24 h. The size and polydispersity at each sampling time were measured by DLS using a short program of six runs of 4 s to be able to highlight aggregation. The position and attenuator were fixed at 4.65 cm and 8, respectively.

### 2.10. Cell Culture

The immortalized human umbilical vein endothelial cell line EA.hy926 (ATCC^®^ CRL-2922™) and the likely human glioblastoma cell line U-87 MG (ATCC^®^ HTB-14™) were purchased from the American Type Culture Collection (ATCC, Manassas, VA, USA). The EA.hy926 were cultured in DMEM-C (37 °C, 5% CO_2_). The U-87 MG cells were cultured in EMEM supplemented by 10% FBS, 100 µg/mL streptomycin and 100 UI/mL penicillin (37 °C, 5% CO_2_).

### 2.11. Morphological Effect of Fisetin on EA.hy926 Endothelial Cells

Exponentially growing EA.hy926 endothelial cells were plated onto 96-wells plates at 5000 cells/well and cultured for 24 h (37 °C, 5% CO_2_), then free fisetin, F3 and F4 liposomal fisetin were added to the wells to reach concentrations ranging from 17 to 350 µM. After 2 h exposure, medium was removed and cells were fixed and colored and observed at a magnification of ×125 using an Olympus IM microscope. Cell morphology was assessed by contouring the cells to obtain morphological parameters using the ImageJ software (National Institutes of Health, Bethesda, MD, USA). Circularity was calculated by the following formula:

Circularity = 4π × area × perimeter^−2^

and the form factor was defined as

Form factor = 1 − circularity

The mean form factor of the control endothelial cells was 0.4. The results were expressed as a percent of the controls using the following formula [11]:

100 × [1 − (circularity of treated cells)/(circularity of control cells)]

### 2.12. Cytotoxicity Assay on EA.hy926 and U 87-MG

Exponentially growing cells EA.hy926 or U 87 MG were plated onto 96-wells plates at 10,000 cells/well and cultured for 24 h (37 °C, 5% CO_2_). Then, free fisetin, free cisplatin, F4 fisetin-loaded liposomes, F4-cisplatin-loaded liposomes, a mixture of F4 cisplatin-loaded and F4 fisetin loaded liposomes (ratio 1:5), F4 co-loaded liposomes and F4 empty liposomes were added to the wells to reach concentrations ranging from 3 to 100 µM for cisplatin and from 18 to 700 µM for fisetin. Control cells were exposed to DMSO or HEPES buffer. Viability was assessed using the MTT ((1-(4,5-dimethylthiazol-2-yl)-3,5-diphenyltetrazolium) test and absorbance was read at 550 nm in a microplate reader 800 TS (Biotek Instrument, Winooski, VT, USA). The results are expressed in percent of viability compared to the same concentration of solvent (DMSO for free fisetin and HEPES buffer for free cisplatin and liposomal suspensions).

To evaluate the potential synergic effect of the combination of cisplatin and fisetin, the combination index (CI) was calculated using the Chou and Talalay equation:

CI = (IC50_F/F_/IC50_F/F+C_) + (IC50_C/C_/IC50_C/F+C_)

where IC50 is the concentration required to kill 50% of the cells, IC50_F/F_ is the IC50 of fisetin in fisetin-loaded liposomes, IC50_F/F+C_ is the IC50 of fisetin in co-loaded liposomes, IC50_C/C_ is the IC50 of cisplatin in cisplatin liposomes and IC50_C/F+C_ is the IC50 of cisplatin in co-loaded liposomes. CI < 0.9 indicates synergism, CI between 0.9 and 1.1 indicates additivity and CI > 1.1 indicates antagonism [39].

### 2.13. Statistics

All measurements are presented as mean ± standard deviation. The number of repetitions is indicated for each experiment. For each test (except for transmission electron microscopy and DSC), three samples of each formulation were taken and measured in triplicate. A non-parametric Mann–Whitney test was used to calculate significant differences; *p*-values less than 0.05 were considered to be significant. All calculations and statistical tests were performed using GraphPad Prism^®^ software (GraphPad Software, San Diego, CA, USA).

## 3. Results and Discussion

The liposomal formulation previously developed by our group for fisetin encapsulation [17] has been chosen as the basic formulation to improve. Preliminary experiments have been performed on blank liposomes to establish the preparation protocol and the lipid compositions of liposomes to investigate in this work (data not shown). Several formulations with increasing cholesterol percentage (from 8.9 to 29.6%mol of cholesterol) have been envisaged to encapsulate cisplatin with fisetin. The influence of the nature of the phospholipid was also studied by using an unsaturated phospholipid, DOPC, or a saturated one, DSCP.

### 3.1. Impact of the Cholesterol Ratio on Fisetin-Loaded and Cisplatin-Loaded Liposomes

Several liposomal formulations encapsulating either fisetin or cisplatin were prepared with an increasing cholesterol ratio, as described in Table 1. F1 formulation (8.9 molar ratio of cholesterol) corresponded to the composition already published in our group [17]. However, the literature review showed that cisplatin-loaded liposomal formulations needed a higher cholesterol ratio to reduce the membrane fluidity and prevent the leakage of cisplatin, from 12 mol% [40] to 46 mol% [41], with most of them between 30 and 40 mol% of cholesterol [33,34,36,42,43,44]. Therefore, to encapsulate both fisetin and cisplatin into the same liposomes, an increase of the cholesterol/DOPC ratio was mandatory in comparison to the basic formulation (F1). F3, F4 and F5 formulations were composed with an increasing molar ratio of cholesterol.

To be able to estimate at this point which formulation to use for the co-encapsulation, the liposomes were purified using a method able to eliminate both free lipophilic and hydrophobic drugs. Fisetin-loaded liposomes and cisplatin-loaded liposomes were then purified using size-exclusion chromatography: liposomes would not be trapped into the sephadex gel, whereas the free drugs would remain in the porous beads. The size, PDI and drug encapsulation of those liposomes are reported in Table 2.

Fisetin encapsulated in the fisetin-loaded liposomes with the higher molar ratio of cholesterol (F6) precipitated the day of preparation and was not considered for further development. There was no difference in fisetin encapsulation in formulations F3 and F4. However, the fisetin-to-lipid ratio (respectively 16.5 and 12.5 mg/g) was relatively lower than the liposomal formulation (F1) developed by Mignet et al. (18 mg/g) [17]. This can be explained by the higher quantity of cholesterol used in F3 and F4 to prevent the leakage of cisplatin outside the liposomes. The results showed that cisplatin encapsulation, i.e., EE and DL, were not significantly divergent considering formulations 3 and 4.

In the literature, encapsulation of cisplatin was higher than in this work [33,34,44,45,46]. However, they used different formulation approaches: increase of the apparent solubility of cisplatin by heating at 80 °C to reach 17 mg/mL [46], use of higher lipid concentration [47], conjugation of the cisplatin to a phospholipid [45], use of a higher cholesterol ratio [33,34,36,44] or use of saturated phospholipids.

### 3.2. Impact of the Saturation of the Phospholipid

Among the strategies described to increase the loading of cisplatin into liposomes, the increase of cholesterol ratio is widely used but could not be envisaged for a co-encapsulation with fisetin. Heating at 80 °C was not considered further as flavonoids can be heat sensitive [48]. Then, a modification of the phospholipid composing the liposomes has been investigated.

Saturated lipids are known to rigidify the lipid bilayer [49] as they have a high transition temperature [50]. The vesicle would always be in the ordered gel phase, where the hydrocarbon chains are fully extended and closely packed. Using a saturated lipid could prevent the leakage of cisplatin from the liposomes. Hence, most of the cisplatin-loaded liposomes reported in the literature are formulated with saturated lipids such as DSPC [51,52,53,54], DPPC [47,54,55] or HSPC [44,56,57]. However, more packed phospholipids could mean less room for fisetin or cholesterol. Considering this, we tried to encapsulate fisetin in liposomes formulated with DSPC and a low (F2) or a high (F5) ratio of cholesterol (Table 1). Both fisetin-loaded F2 and F5 led to a very low drug loading (0.23%) with precipitated fisetin remaining in the preparation batch. Moreover, the preparation process needed to be at 65 °C (temperature above the gel-liquid crystalline phase transition, 55 °C), and fisetin is known to be degraded by temperature in aqueous solution [58]. Therefore, we chose not to proceed with those formulations.

### 3.3. Impact of the Co-Encapsulation on the Encapsulation of Each Drug into Liposomes

Formulations 3 and 4 were chosen for the co-encapsulation of fisetin and cisplatin. The size, PDI and encapsulation of those liposomes are reported in Table 2. The particle sizes of all formulations were below 200 nm with polydispersity below 0.2. Liposomes were spherical according to TEM pictures (data not shown). The co-encapsulation of cisplatin and fisetin evidenced drug loading of, respectively, 0.8 ± 0.1% and 1.6 ± 0.3% for formulation 3 (cholesterol molar ratio of 17.0%) and 0.8 ± 0.1% and 1.7 ± 0.3% for formulation 4 (cholesterol molar ratio of 20.8%). There was no significant difference in terms of fisetin or cisplatin encapsulation between both formulations or between single-drug encapsulation or co-encapsulation. Consequently, co-encapsulation does not affect the encapsulation of each drug. At this stage, liposomes co-encapsulating cisplatin and fisetin could be prepared with a drug weight ratio of 1:2 and a cholesterol ratio between 17.0 and 20.8 mol% with success. It is of note that the amounts of cisplatin and fisetin loaded into these liposomal formulations are promising for their further in vitro biological effect.

### 3.4. Stability Over Time of the Liposomes

**Figure 1:**
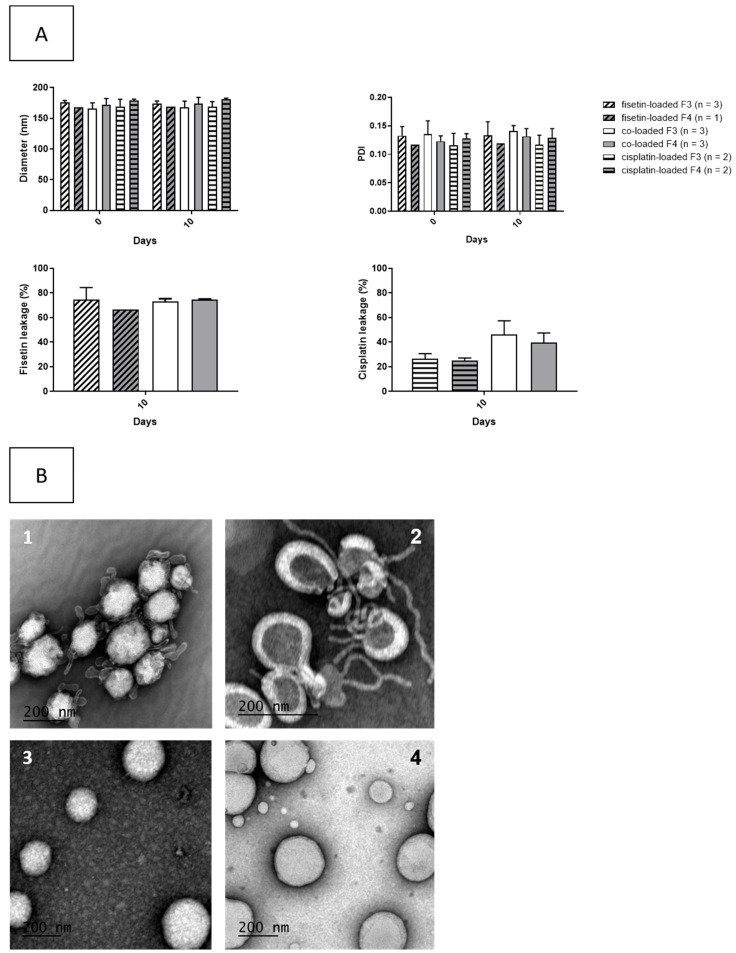
Stability of the liposomal formulations. (**A**) stability of size, PDI and drug leakage of the different liposomal formulations over 10 days (mean ± SD). (**B**) TEM of the liposomal formulations co-encapsulating cisplatin and fisetin (1: formulation 3 and 2: formulation 4) and liposomal formulation encapsulating only fisetin (3) or only cisplatin (4) after three days of storage.

Liposomal formulations were stored at 5 ± 3 °C and purified again after 10 days. Size, PDI and drug concentration remaining into liposomes were assayed. The results are presented in Figure 1A. There was no modification of size and PDI over time; however, leakage of the encapsulated drugs was evidenced. After 10 days of storage, the percentage of fisetin leakage was similar for the fisetin-loaded liposomes and in the co-encapsulated liposomes in formulation F3 containing 17% cholesterol (74.5 ± 9.9% for fisetin-loaded F3, 72.9 ± 2.3% for co-loaded F3), while it seemed different in formulation F4 containing 20% cholesterol (66.4% for fisetin-loaded F4 and 74.5 ± 0.6% for co-loaded F4). The increase of cholesterol content seems to influence the leakage of the fisetin. Moreover, the loss of cisplatin seemed higher when the two drugs were co-encapsulated than when it was encapsulated alone (26.6% ± 4.0% for cisplatin-loaded F3 versus 46.2% ± 11.2% for co-loaded F3, 24.8% ± 2.2% for cisplatin-loaded F4 versus 39.8% ± 7.6% for co-loaded liposomes F4). This could be explained by a disruption of the bilayers induced by the fisetin encapsulation into the bilayer: indeed, TEM experiments evidenced the disruption of the lipid bilayer, such as blebbing or tabulation, three days after the preparation (Figure 1B). Those disruptions were observed only in liposomes encapsulating both drugs.

### 3.5. Study of the Influence of Fisetin Incorporation into the Lipid Bilayer

To further understand the leakage of drugs from liposomes, we investigated the influence of the insertion of fisetin into the lipid bilayer.

Fisetin is a lipophilic compound as its octanol-water partition coefficient is estimated at 3.2 [17]. It is consequently presumed to be located into the lipid bilayer of the liposomes. Mignet et al. determined that the maximum ratio of fisetin (3.2 wt%) was observed within liposomes containing 4 wt% (8.9 mol%) of cholesterol [14,17]. Fisetin is incorporated inside the lipid bilayer, with its non-polar part inside the hydrophobic bilayer and the more polar ones forming hydrogen bonds with the polar head groups of the phospholipids [16,59]. This comportment is similar to cholesterol and Mohapatra et al. showed a cholesterol-induced expulsion of fisetin from dimyristoylphosphatidylcholine liposome membrane [59]. Consequently, we investigated the influence of the cholesterol/DOPC ratio on the incorporation of fisetin into the lipid bilayer. DSC has been previously used to determine the maximum incorporation ratio of paclitaxel [60] and lidocaine [61] into the lipid bilayer using the main transition temperature and the enthalpy of the main phospholipid composing liposomes. In this work, DSC experiments were performed by analyzing the melting event of DOPC as a function of the addition of the other bilayer components.

As it is well known that a high ratio of cholesterol can lead to the disappearance of the endothermic peak of neighboring phospholipids [62,63,64,65], we first investigated the maximum cholesterol ratio for which the DOPC peak could still be observed (Figure S1). DOPC alone exhibited a main transition peak at −21 °C. It should be noted that a peak at Tonset −16 °C appeared when DODA-GLY-PEG2000 was introduced in the liposomal formulation. Liposomes with increasing cholesterol ratio were prepared at a fixed initial mass ratio of fisetin of 3.2%. Figure S1 shows a decreasing onset temperature of the DOPC main transition peak (from −21.0 to −26.1 °C) and a decreasing enthalpy of this thermal event (from 21,663 to 535 J/mol) with the increase of the cholesterol ratio, as expected. With a cholesterol ratio of 26.7 mol%, the DOPC endothermic peak is almost completely removed, and at a higher ratio, this peak is undetectable. We then checked that fisetin in suspension did not affect the melting event of DOPC by adding a fisetin suspension to empty liposmes. When fisetin was precipitated, sampling the supernatant did not modify the Tonset of DOPC.

**Figure 2:**
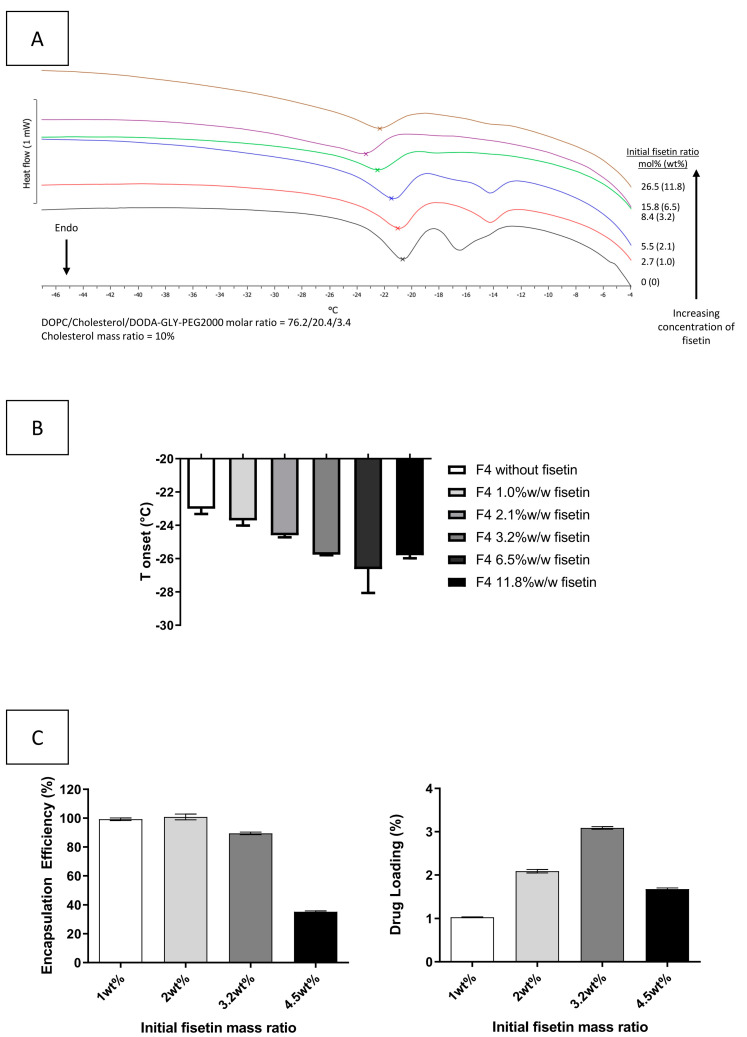
Influence of fisetin insertion into the lipid bilayer. (**A**) Representative DSC thermograms of the melting of DOPC contained in the lipid bilayer of liposomes with increasing amount of fisetin at cholesterol mass ratio of 10%, n = 3. (**B**) Modification of the Tonset of the DOPC melting in function of the proportion of fisetin inserted (mean ± SD, n = 2). (**C**) Encapsulation efficiency and drug loading of fisetin-loaded liposomes with increasing amount of fisetin incorporated into the formulation (mean ± SD, n = 2).

We consequently investigated the influence of increasing the initial fisetin ratio on a lipid bilayer with 20.4 mol% (10 wt%, F4, Table 1) of cholesterol using DSC. Figure 2A,B show that the onset temperature of the DOPC transition decreases from −23.3 to −25.8 °C with the increase of the initial fisetin ratio from 0 to 3.2 wt%; with a higher initial ratio of fisetin, the onset temperature of the DOPC transition remains stable around −25.8°C. The decrease in the transition onset temperature demonstrates the incorporation of the fisetin into the lipid bilayer. As a fisetin ratio higher than 3.2 wt% showed no further disturbance, the maximum amount of fisetin incorporated into the lipid bilayer can be estimated around 3.2 wt%.

Then, fisetin-loaded liposomes (F4, Table 1) with an increasing initial ratio of fisetin, from 1 to 4.5 wt% were prepared for determining the fisetin encapsulation by HPLC dosage. As the solubility of fisetin in water was previously experimentally estimated as inferior to 5 µg/mL, these fisetin-loaded liposomes were purified by a simple filtration to remove non-encapsulated and precipitated fisetin. Figure 2C shows that at 1 and 2 wt% of initial ratio, all of the fisetin is encapsulated into the liposomes, but at 3.2 wt%, only 90% is encapsulated. However, the DL is the highest for 3.2 wt%, suggesting that the best loading of fisetin into the liposomes is obtained with a 3.2 wt% initial ratio of fisetin. A higher initial ratio of fisetin led to a drastic decrease of the EE and DL, implying a disturbance of the lipid bilayer by the too large proportion of fisetin. Those results confirm DSC results with a maximum incorporation of fisetin into the lipid bilayer around 3.2 wt% (20.4 mol% of cholesterol), which is the same as determined by Mignet et al. for a lower content of cholesterol (F1, 8.mol% of cholesterol) [17]. To conclude, the cholesterol ratio in the range studied does not seem to have a significant impact on the ability of the lipid bilayer to incorporate fisetin, but the quantity of fisetin added in the preparation has an impact. Fisetin must interact or be in competition with another lipid.

**Figure 3:**
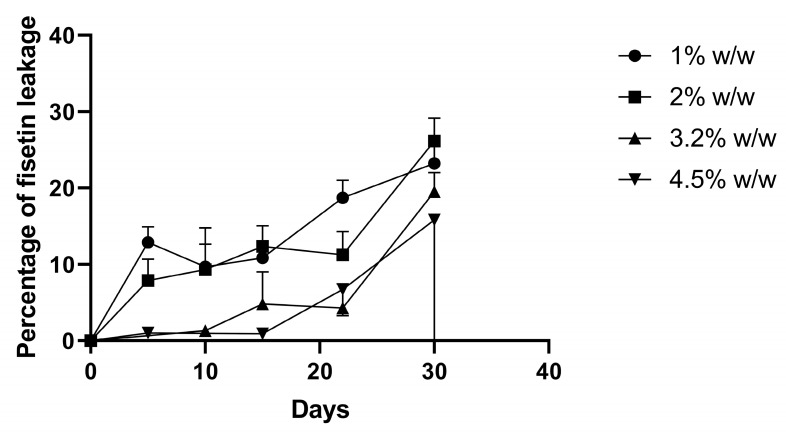
Stability of fisetin-loaded liposomes F4 containing 1, 2, 3.2 or 4.5 wt% of cholesterol (mean ± SD, n = 2). Liposomes were purified using filtration to remove non-encapsulated and precipitated fisetin at each sampling time.

We then hypothesized that even if the insertion ability of fisetin into the lipid bilayer was the same with 8.9 mol% (F1) or 20.4 mol% (F4) of cholesterol, maybe the higher cholesterol ratio combined with a high ratio of fisetin induced a destabilization of the lipid bilayer over time, leading to the expulsion of fisetin. To confirm this hypothesis, four different fisetin-loading liposomes with 1 wt%, 2 wt%, 3.2 wt%, or 4.5 wt% were prepared and purified using filtration. The leakage of fisetin was followed over 30 days (Figure 3). However, encapsulating less fisetin did not lead to a better stability of the fisetin encapsulation. Moreover, the leakage of fisetin was lower using filtration purification (<10% for F4 after 10 days) than using Sephadex purification (74.5% for F4, Figure 1A). The impact of the purification was already seen at D0: purification by size-exclusion chromatography led to a clear loss of fisetin: DL of 1.3% for F4 in Table 2 compared to DL above 3% for F4 purified by filtration (Figure 2C).

Sephadex purification might not be the more appropriate purification method due to its potential destabilization of the membrane containing fisetin.

An alternative purification method was envisaged: elimination of free cisplatin by ultracentrifugation, followed by the suspension of the pellet in fresh buffer with a finishing step consisting in the elimination of the free non-water-soluble fisetin by filtration. However, ultracentrifugation led to filter plugging and could not be used; thus, the alternative purification process could not be completed. Purification by dialysis and filtration has also been envisaged and started to show promising results (Figures S2 and S3).

We observed a limited stability of liposomes, as expected. Indeed, liposomal formulations in clinical use are often presented in a freeze-dried form [66]. For example, CPX-351 has a stability of 4 h after reconstitution (summary product characteristic). Moreover, the stability of the previously published liposomal cisplatin was rarely studied. Shein et al. described a loss of around 50% of cisplatin in two months (less than 6% in one week) [36], and Toro-Cordova et al. reported a loss around 10% of cisplatin leakage after 30 days [44]. However, the process of the purification step after analysis at due time is not described. We can wonder if there is an underestimation of the cisplatin leakage. To improve the stability of our formulations throughout time, freeze-drying of the liposomes would be further investigated. Fresh liposomal formulations F4 were chosen to be used for the in vitro evaluation.

### 3.6. Release Study of the Co-Loaded Formulation 4 in PBS

**Figure 4:**
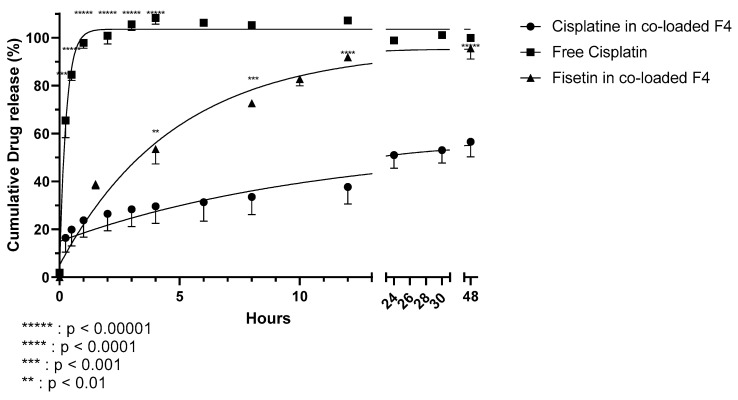
In vitro release of cisplatin and fisetin by formulation 4 co-encapsulating cisplatin and fisetin in PBS. Liposomal cisplatin (circle), free cisplatin (square) and liposomal fisetin (triangle) (mean ± SD, n = 3).

Before assaying the efficacy of our formulation on cell lines, we explored its capacity to release fisetin and cisplatin in vitro. An in vitro release study in PBS at 37 °C of F4 co-loaded liposomes was conducted by dialysis to evidence a release of the two drugs from the liposomes. The in vitro release of cisplatin from the liposomes is compared with free cisplatin in Figure 4.

A biphasic release was observed in both cases with a fast release up to one hour. After one hour, almost all the free cisplatin was released from the dialysis bag versus only 23.8 ± 7.1% for encapsulated cisplatin. After one hour, a sustained release of cisplatin from the liposomes was observed, to reach 56.6 ± 6.3% of cisplatin release after 48 h. After 15 min, a difference between the release of free cisplatin and encapsulated cisplatin was observed and remained significant for 48 h. This delayed release of encapsulated cisplatin is expected. The release of fisetin from the liposomes could not be compared to free fisetin as it is not soluble in water and solubility is under the limit of quantification of our dosage method. However, Figure 4 shows that the release of fisetin is significantly higher than the release of cisplatin, with 91.9 ± 0.2% and 95.5 ± 4.4% of release after 12 h and 48 h, respectively. This difference of both drug releases could have an influence on their in vivo combinatory effect.

### 3.7. Colloidal Stability and Release Study in Culture Medium of Co-Loaded Liposomes F4

**Figure 5:**
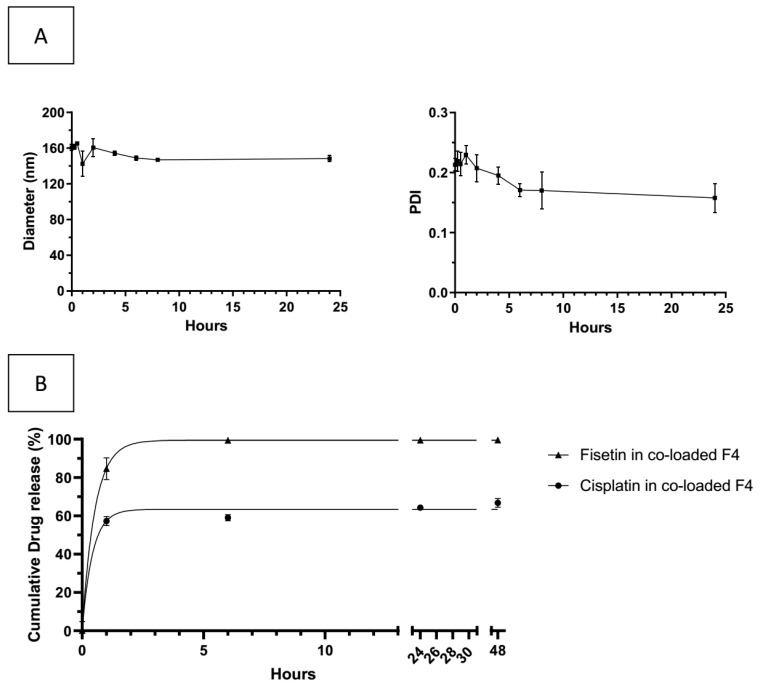
Stability of the formulation 4 co-encapsulating cisplatin and fisetin in complete culture medium (mean ± SD, n = 3). (**A**) stability of size and PDI. (**B**) In vitro release of cisplatin and fisetin by formulation 4 co-encapsulating cisplatin and fisetin in DMEM-C: liposomal cisplatin (circle) and liposomal fisetin (triangle).

In order to anticipate the release in physiological medium, the behavior of the co-loaded liposomes F4 has been investigated in the cell culture medium. As shown in Figure 5A, the size and PDI of the formulation was stable for 24 h in DMEM-C. Figure 5B indicates the release of cisplatin and fisetin from the co-loaded liposomes F4 in DMEM-C at 37 °C. Fisetin and cisplatin are released more quickly in the culture medium than in PBS, with a burst release during the first hour followed by a sustained release to reach 100% of release in 6 h for fisetin and 67% of release in 48 h for cisplatin. This may be explained by the binding of fisetin to serum protein with a better affinity than liposomes [67]. Increasing the viscosity of the aqueous core with an hydrophilic polymer could be an alternative to prevent the burst release of cisplatin [68,69,70]. Moreover, the difference of release kinetics between both drugs observed in PBS is confirmed in culture medium. These data would be considered to discuss the cellular effect of liposomal formulations.

Liposomes F4 co-encapsulating fisetin and cisplatin were able to release the drugs into the medium and were then incubated with EA.hy 926 cells to evaluate their antiangiogenic effect and with U 87-MG cells to evaluate their cytotoxic effect. As cisplatin is toxic for EA.hy 926, the antiangiogenic effect was only evaluated on fisetin-loaded liposomes.

### 3.8. Effects of Fisetin on Endothelial Cells EA.hy 926

**Figure 6:**
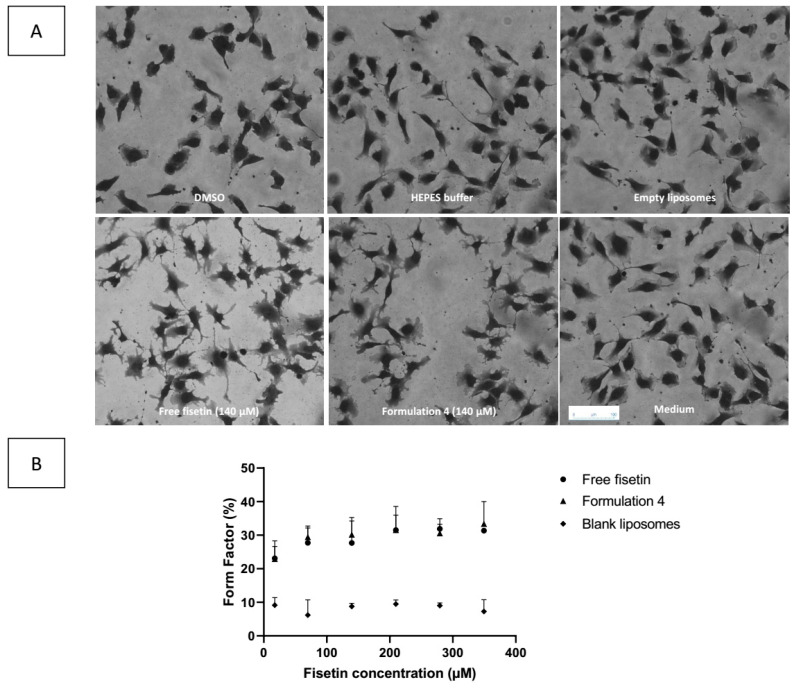
Effect of free fisetin and liposomal fisetin on endothelial Ea.hy 926 cell line (mean ± SD, n = 4). (**A**) modification of EA.hy 926 cells’ morphology (magnification ×125). (**B**) form factor of empty liposomes (diamond), free fisetin (circle) and fisetin-loaded formulation 4 (triangle).

As an anti-angiogenic agent, fisetin is known to have a morphological effect on endothelial cells after 2 h of exposure at non-toxic concentration, and exert a cytotoxic effect after 24 and 48 h of exposure [11]. Therefore, we incubated the fisetin-loaded liposomes F4 on EA.hy 926 cells to assess if liposomal fisetin had the same effect. Figure 6A depicts the EA.hy 926 cells after 2 h of exposure to free fisetin (control: DMSO), fisetin-loaded F4, empty liposomes F4 (control: HEPES buffer). Endothelial cells exposed to empty liposomes, HEPES buffer and DMSO are similar to the control endothelial cells, whereas endothelial cells exposed to fisetin free or loaded into liposomes F4 show some cell extensions. To quantify those morphological modifications, contouring of the cells was performed using ImageJ software and the form factor was determined: the form factor was the same for fisetin-loaded liposomes F4 than for free fisetin (Figure 6B). The fact that the morphological effect after two hours of exposure was similar between free and liposomal fisetin was expected because the release of fisetin in the culture medium was quick—85% after one hour and almost 100% after 6 h. The liposomal encapsulation does not affect the integrity and the efficacy of fisetin.

Moreover, there was a significant decrease in concentration required to kill 50% of the cells (IC50) between free fisetin and the fisetin-loaded liposomes F4 after 24 h of exposure and a similar trend was observed after 48 h (Table 3). Those results suggest that the encapsulation of fisetin slightly increased its toxic activity on EA.hy 926 cells by favoring cell penetration. It also important to note that liposomal encapsulation of fisetin enables the fisetin to reach this efficient concentration without the use of an organic solvent (DMSO).

### 3.9. In Vitro Cytotoxicity Assay on Glioblastoma Cells U 87-MG

Cisplatin alone can exhibit a high cytotoxicity at a low concentration on the U 87-MG cell line [19]. To be able to discriminate the effect of fisetin, the in vitro cytotoxicity assay on glioma cells has been performed using a fisetin:cisplatin ratio 5:1 *w*/*w*.

**Figure 7:**
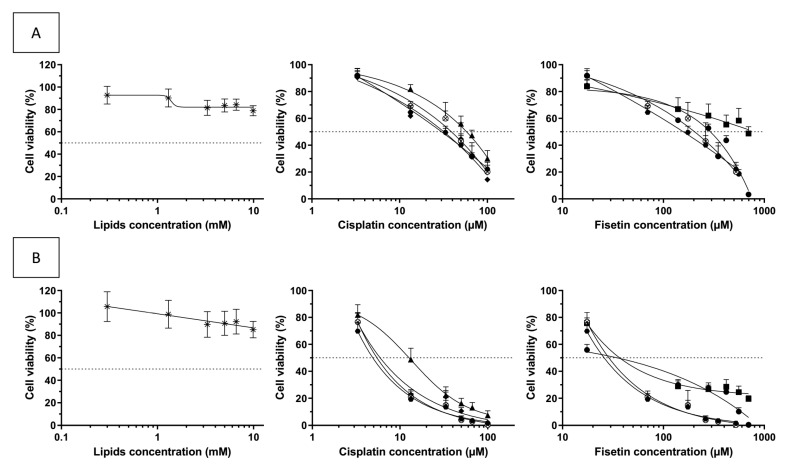
Comparison of the cytotoxicity of empty liposomes (star), free fisetin (circle), free cisplatin (diamond), fisetin-loaded liposomes (square), cisplatin-loaded liposomes (triangle), mixture of fisetin-loaded liposomes and cisplatin-loaded liposomes (cross in circle) and liposomes co-encapsulating fisetin and cisplatin (hexagone) after 24 h (**A**) or 48 h (**B**) of exposure (mean ± SD, n = 5).

A standard colorimetric MTT assay was used to investigate the antiproliferative effect of free cisplatin, free fisetin, a mixture of fisetin-loaded liposomes F4 and cisplatin-loaded liposomes F4 to simulate the co-administration of single agent-loaded liposomes, co-loaded liposomes F4 and empty liposomes F4. The results are reported in Figure 7 and Table 4. Empty liposomes showed no cytotoxic effect on the U 87-MG cell line. The IC50 of the U 87-MG cells was significantly higher for cisplatin-loaded liposomes F4 than for free cisplatin (15 ± 8 µM versus 6 ± 3 µM after 48 h of exposure, *p* < 0.05). Those results were expected because of the sustained release of cisplatin from the liposomes. Fisetin also exhibits a cytotoxic effect on the U 87-MG cell line with an IC50 of 44 ± 32 µM after 48 h. There was no difference between the cytotoxicity of free fisetin and fisetin-loaded liposomes F4. It worth noting that the addition of fisetin enables the mixture of the liposomes and the co-loaded liposomes F4 to reach the same cytotoxic activity as free cisplatin. Therefore, this co-loaded liposomal formulation appears promising for further antitumoral activity experiments on animals, expecting a better tumoral accumulation and a lower toxicity of the liposomal form compared to free cisplatin.

As the cytotoxicity of fisetin against U 87-MG was observed, we explored the potential synergism between fisetin and cisplatin on the cytotoxicity against U 87-MG using the Chou–Talalay equation. The combination index determined was 1.1 for 24 h and 48 h of exposure and, highlighting additivity [39], indicating that the ratio between fisetin and cisplatin could be optimized. However, fisetin was chosen for its antiangiogenic effect and the combination of the two drugs must be studied in vivo.

As there is no controlled ratio of released drug and no synergism at the fisetin:cisplatin ratio chosen, one may wonder if this co-encapsulation presents an interest and whether co-administration would be better as it allows flexibility in the drugs ratio and timing of injection. It would be interesting to test other fisetin:cisplatin ratios and to compare co-encapsulating liposomes with the co-administration of the two drugs both encapsulated in an optimal nanocarrier formulation for each drug. Markowski et al. evidenced that paclitaxel and doxorubicin conjugated to PGA nanoparticles significantly increased the tumor inhibition rate on MDA-MB-231 tumor-bearing mice in comparison to a mixture of individually conjugated drugs or the combination of free drugs [71]. However, few studies reporting co-encapsulation have compared it to co-administration. It appears necessary to perform this comparison in vivo.

## 4. Conclusions

Thanks to DSC experiments, a compromise in the cholesterol content in the liposomal formulation has been found to insert the maximum of fisetin into the lipid bilayer and a sufficient proportion of cholesterol to maintain sufficient cisplatin amounts over 10 days. The purification method has been identified as a critical parameter of the preparation process. The limited stability of the drug encapsulation into liposomes appeared to be related to the choice of purification method. An optimized formulation co-encapsulating fisetin and cisplatin at concentrations able to exert the double activity against the targets was successfully designed, and its stability could be improved by freeze-drying, as is used in many formulations already in use. This co-loaded formulation was able to retain the activity of fisetin on endothelial cells and was effective against glioblastoma cells. We report, for the first time to our knowledge, a cytotoxic activity of fisetin on glioblastoma U 87-MG cells. These results open the door for further in vivo experiments to investigate a new strategy for cancer therapy.

## Figures and Tables

**Table 1:** Lipid formulations of the liposomes assayed (molar ratio).

Composition	F1	F2	F3	F4	F5	F6
DOPC	87.0	-	79.0	75.3	-	66.7
DSPC		85.8	-	-	75.3	-
Cholesterol	8.9	6.8	17.0	20.8	21.0	29.6
DODA-GLY-PEG2000	4.1	7.4	4.0	3.9	3.7	3.7

**Table 2:** Size, PDI and drug encapsulation of fisetin-loaded, cisplatin-loaded and fisetin and cisplatin-co-loaded liposomes (mean ± SD).

Formulation	Diameter (nm)	PDI	EE (%) Fisetin	DL (%) Fisetin	Drug to Lipids Ratio (mg/g) Fisetin	EE (%) Cisplatin	DL (%) Cisplatin
Fisetin-Loaded Liposomes							
Formulation 3 (n = 3)	176 ± 3	0.13 ± 0.02	49.5 ± 21.2	1.6 ± 0.7	16.5 ± 7.0		
Formulation 4 (n = 2)	176 ± 11	0.14 ± 0.04	37.8 ± 0.3	1.3 ± 0.1	12.8 ± 0.1		
Formulation 6 (n = 2)	175 ± 8	0.12 ± 0.01	30.5 ± 13.7	1.0 ± 0.5	10.5 ± 5.4		
Cisplatin-loaded liposomes							
Formulation 3 (n = 2)	169 ± 12	0.12 ± 0.02				14.6 ± 3.4	0.8 ± 0.2
Formulation 4 (n = 2)	179 ± 2	0.13 ± 0.01				12.9 ± 1.1	0.7 ± 0.1
Formulation 6 (n = 1)	175	0.14				13.7	0.7
Co-loaded liposomes							
Formulation 3 (n = 4)	166 ± 8	0.14 ± 0.02	47.8 ± 6.3	1.6 ± 0.3	16.2 ± 2.9	13.7 ± 2.7	0.8 ± 0.1
Formulation 4 (n = 5)	173 ± 8	0.12 ± 0.01	50.3 ± 6.8	1.7 ± 0.3	16.8 ± 2.5	14.5 ± 1.5	0.8 ± 0.1

**Table 3:** IC50 values (µM) as function of fisetin on EA.hy 926 cell line (mean ± SD, n = 4). * *p* < 0.05 versus free drug.

IC50 (µM)		
Time of Exposure	Free Fisetin	Formulation 4
24 h exposure	183 ± 18	135 ± 10 *
48 h exposure	131 ± 6	99 ± 8

**Table 4:** IC50 values (µM) as function of cisplatin and fisetin on U-87 MG cell line (mean ± SD, n = 5). * *p* < 0.05 versus free drug. ^$^ *p* < 0.05 versus liposomal drug encapsulated alone.

IC50 (µM)							
Time of Exposure	Active Substance	FreeCisplatin	Free Fisetin	Cisplatin-Loaded Liposomes F4	Fisetin-Loaded Liposomes F4	Mixture of Fisetin-Loaded and Cisplatin-Loaded Liposomes F4	Liposomes F4 Co-Encapsulating Fisetin and Cisplatin
24 h exposure	Cisplatin	34 ± 12	-	58 ± 23	-	42 ± 23	36 ± 20
	Fisetin	-	300 ± 77	-	366 ± 305	215 ± 119	190 ± 103
48 h exposure	Cisplatin	6 ± 3	-	15 ± 8 *	-	7 ± 2 ^$^	6 ± 2 ^$^
	Fisetin	-	44 ± 32	-	45 ± 30	35 ± 11	32 ± 10

## Data Availability

The data presented in this study are available on request from the corresponding author.

## Supplementary Materials

The following are available online at https://www.mdpi.com/article/10.3390/pharmaceutics13070970/s1, Figure S1: results representative of different DSC experiments of liposomes with increasing ratio of cholesterol (from 0 to 13 wt%), which were carried out in duplicate; cholesterol ratios of 20 and 30% led to an undetectable main transition, and thus data are not shown. Figure S2: shelf stability of fisetin-loaded liposomes F3 over 30 days of storage; black shows results after size-exclusion chromatography purification and white shows results after filtration purification (n = 2). Figure S3: shelf stability of cisplatin-loaded liposomes F3 and F4 over 30 days of storage; black shows results after size-exclusion chromatography purification and white shows results after dialysis purification (n = 2).
