# Supplementary material for: Co-Encapsulation of Fisetin and Cisplatin into Liposomes for Glioma Therapy: From Formulation to Cell Evaluation
Source: Pharmaceutics. 2021 Jun 26;13(7):970. doi: 10.3390/pharmaceutics13070970 (PMC8309049; doi:10.3390/pharmaceutics13070970)
Supplement: Supplementary file 1 [file pharmaceutics-13-00970-s001.zip › pharmaceutics-1258923-supplementary.pdf]

pharmaceutics

MDPI

Supplementary Materials: Co-encapsulation of fisetin and cisplatin for glioma therapy: from formulation to cell evaluation

Morgane Renault-Mahieux, Victoire Vieillard, Johanne Seguin, Philippe Espeau, Dang Tri Le, René Lai-Kuen, Nathalie Mignet, Muriel Paul and Karine Andrieux

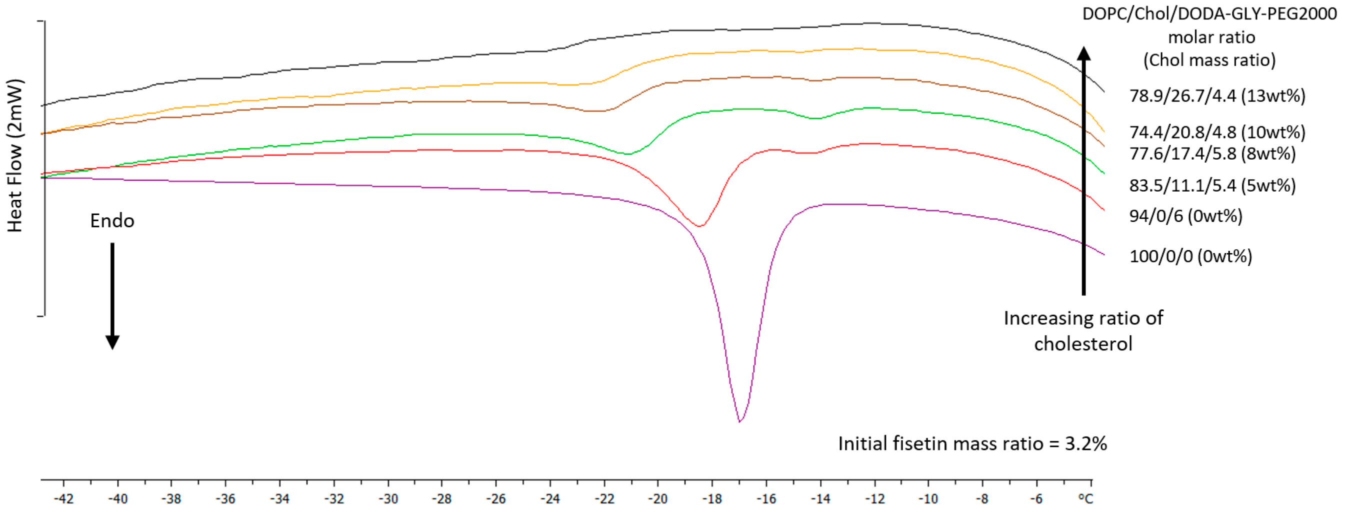

Figure S1. Results representative of different DSC experiments of liposomes with increasing ratio of cholesterol (from 0 to 13 wt%), which were carried out in duplicate; cholesterol ratios of 20 and 30% led to an undectable main transition and thus datas are not shown.

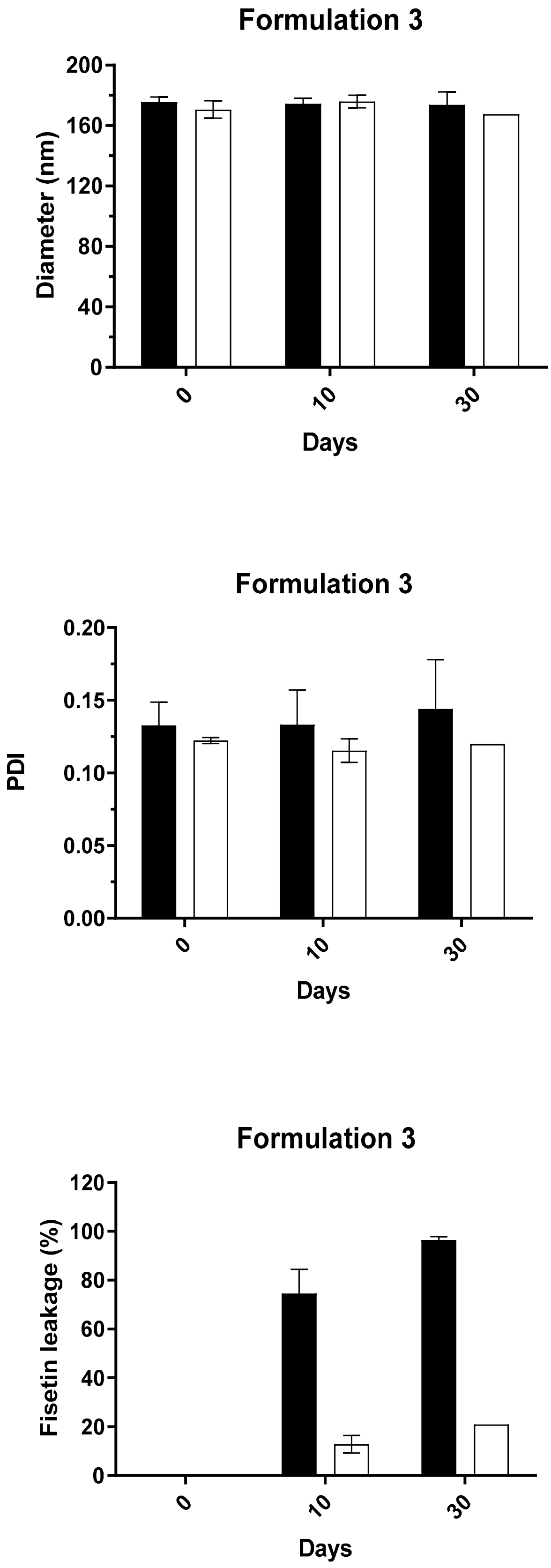

Figure S2. Shelf stability of fisetin-loaded liposomes F3 over 30 days of storage. Black shows results after size-exclusion chromatography purification and white shows results after filtration purification (n = 2).

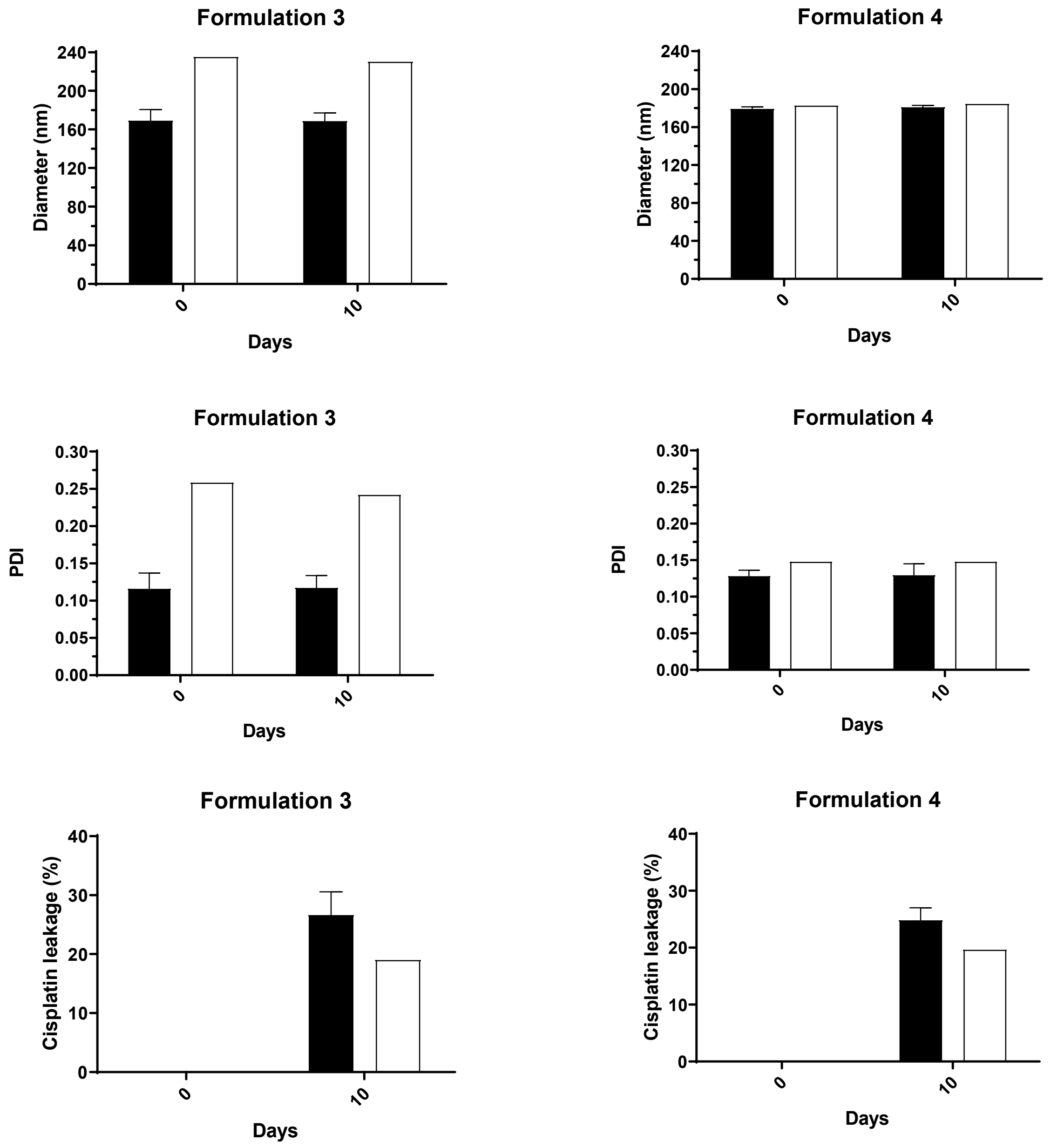

Figure S3. Shelf stability of cisplatin-loaded liposomes F3 and F4 over 30 days of storage. Black shows results after size-exclusion chromatography purification and white shows results after dialysis purification (n = 2).
